# Antibiotic susceptibility patterns of pathogens isolated from hospitalized patients with advanced HIV disease (AHD) in Bihar, India

**DOI:** 10.1093/jacamr/dlad151

**Published:** 2024-01-02

**Authors:** Vikash Kumar, Shreyas Murali, Jacob Goldberg, Beatriz Alonso, Laura Moretó-Planas, Anthony Reid, Amit Harshana, Sakib Burza, Raman Mahajan

**Affiliations:** Operational Centre Barcelona-Athens, Médecins Sans Frontières, New Delhi, India; Operational Centre Barcelona-Athens, Médecins Sans Frontières, Patna, India; Medical Department, Médecins Sans Frontières, London, UK; Medical Department, Médecins Sans Frontières, London, UK; Operational Centre Barcelona-Athens, Médecins Sans Frontières, Barcelona, Spain; Opérationnel Research Unit, Médecins Sans Frontières, Luxembourg, Luxembourg; Operational Centre Barcelona-Athens, Médecins Sans Frontières, New Delhi, India; Operational Centre Barcelona-Athens, Médecins Sans Frontières, New Delhi, India; London School of Hygiene and Tropical Medicine, London, UK; Operational Centre Barcelona-Athens, Médecins Sans Frontières, New Delhi, India; Care and Public Health Research Institute, Maastricht University, Maastricht, The Netherlands

## Abstract

**Objectives:**

To describe the prevalence of common bacterial pathogens and antibiotic susceptibility patterns amongst advanced HIV disease (AHD) patients admitted between May 2019 and March 2021 to a Médecins Sans Frontières (MSF)-supported AHD inpatient unit in Bihar, India.

**Methods:**

A retrospective analysis of routinely collected demographic, clinical and microbiological data. Antibacterial susceptibility testing was done by an accredited referral laboratory using the modified Kirby–Bauer disc diffusion method.

**Results:**

A total of 238 isolates from 577 patients were identified through culture testing. Patient median (IQR) age was 38 (31–45) years, and 75% were male. Predominant sample types included blood (600; 38%), urine (266; 17%) and sputum (178; 11%). Of the isolated bacteria, *Escherichia coli* (80; 13.9%) was the most prevalent, followed by *Klebsiella pneumonia* (54; 9.4%), *Pseudomonas aeruginosa* (22; 3.8%), *Klebsiella oxytoca* (10; 1.7%), *Proteus mirabilis* (9; 1.6%), and *Acinetobacter baumannii* (7; 1.2%). The resistance pattern showed that most bacterial isolates were highly resistant to commonly prescribed antibiotics such as third-generation cephalosporins, fluoroquinolones and co-trimoxazole. Most pathogens were moderately resistant to antibiotics from the WHO Watch group, such as meropenem and piperacillin/tazobactam. In contrast, isolates were more susceptible to aminoglycosides, such as amikacin, gentamicin and nitrofurantoin.

**Conclusions:**

In Bihar, inpatients with AHD displayed a concerning array of antibiotic-resistant infections. This study provides a starting point from which further work on antimicrobial resistance in this vulnerable cohort of patients can be conducted.

## Introduction

Recent estimates show India is home to 2.3 million people living with HIV (PLHIV).^1^ According to current evidence, approximately one-third of PLHIV had advanced HIV disease (AHD), defined as CD4 count less than 200 cells/mm^3^ and/or WHO clinical stage III or IV disease and/or all children living with HIV under the age of 5 years.^2^ Sepsis, defined as a life-threatening organ dysfunction caused by a dysregulated host response to infection or severe bacterial infections, is the main contributor to high mortality and morbidity in patients with AHD.^3^ A mortality rate of 37% has been reported among AHD patients from a Médecins Sans Frontières (MSF) hospital in Kinshasa in the Democratic Republic of Congo (DRC), and of those, 31% died within 48 h of admission with bacterial infections as a provisional diagnosis.^4^ According to a recent meta-analysis, bacterial infections were the cause of one-third of all hospital admissions and one-quarter of all fatalities in PLHIV.^5^ Another study from Nigeria found elevated mortality and morbidity in AHD cases, likely due to the inability to mount an adequate immune response reflected by a low CD4 count.^3^

Patients with AHD frequently arrive in hospitals in India at a late stage of the disease, typically with sepsis.^6^ The clinician’s priority is therefore the urgent care of sepsis, which typically requires the initiation of immediate antibiotic therapy. In these scenarios, clinicians often do not have time to wait for the results of antibiotic susceptibility testing (AST) and cultures that might identify pathogens and guide appropriate antibiotic use. Therefore, it is customary to use the general Indian National Antibiotic Guidelines for empirical treatment rather than those designed specifically for AHD patients, which are not available in India.^7^

In India, as elsewhere, pathogen prevalence and antibiotic sensitivity patterns are highly variable according to context.^8^ Having accurate knowledge of those patterns would support choosing the most appropriate antibiotic for patients whose immune systems are seriously compromised. With this information, clinicians could start antibiotic treatment more confidently, knowing that the choice was likely to be effective. It is also important to note that most hospitals in the Indian public health setting do not have access to AST. Therefore, having evidence for prevalence and sensitivity profiles of local pathogens could be used to develop guidelines at the local or even state level. Another critical aspect is that AHD patients often arrive at the hospital having already received one or multiple types of antibiotic therapy since they tend to have been unwell for an extended period of time and sought care from multiple providers.

There is a dearth of data on antibiotic susceptibility patterns among PLHIV. One study from the USA showed that MDR Enterobacterales colonization is common in PLHIV.^9^ Another study from the same country indicated that PLHIV had consistently greater rates of antibacterial resistance among Enterobacterales than their immunocompetent counterparts.^10^ One study from Cameroon found high prevalence of MDR and ESBL-producing Enterobacterales isolated from carriage samples among HIV-infected women.^11^

To the authors’ knowledge, a description of the bacterial pathogens and antibiotic susceptibility patterns in AHD patients in India has not yet been described. In response to the need for holistic inpatient care for AHD in Bihar, MSF supports a 36 bed advanced HIV ward in the Guru Gobind Singh Hospital (GGSH) in Patna, Bihar. This retrospective study described the demographic and clinical characteristics, the prevalence of bacterial infections, and antibiotic susceptibility patterns in AHD inpatients receiving care at this facility.

## Material and methods

### Study design and setting

A retrospective observational study was conducted using routinely collected demographic, microbiological and clinical data from the MSF-supported AHD treatment ward within GGSH hospital in Patna, Bihar. Bihar is one of the most populous and socioeconomically challenged states in India. As per the National Aids Control Organisation (NACO), Bihar’s HIV prevalence among people aged 15 to 49 years was 0.16% in 2021, and the state is home to an estimated 142 793 PLHIV. MSF provides a comprehensive care package in this ward that includes free diagnostics, treatment, referral, mental health and palliative care for all patients with AHD. Most AHD patients in this study were referred to the MSF clinic by government-supported ART centres across Bihar, or self-referred. These ART centres are mandated to provide free ART treatment, CD4 and viral load testing, and drugs for the treatment of opportunistic infections. However, ruptures are common in both the availability of diagnostic tests for opportunistic infections and prophylactic drugs. MSF provides a comprehensive care package at its specialized centre for managing patients with AHD. Similar to all MSF projects, care is completely free, including diagnostics, treatment, referral, mental health and palliative care. The diagnostic algorithm for opportunistic infections included the following tests: CD4 count and HIV viral load tests, TB LAM, point of care ultrasonography (POCUS), GeneXpert MTB/RIF for TB, fundoscopy for cytomegalovirus, CrAg^®^ LFA for testing cryptococcal antigen, RK-39 rapid test, and splenic or bone marrow aspiration for visceral leishmaniasis where indicated.

Similar to most public secondary-level hospitals in India, GGSH lacks in-house AST capacity, and therefore all clinical samples were sent to a private fully accredited laboratory for culture and AST.

### Study population and period

The study population included all AHD patients admitted to the MSF-supported AHD ward at GGSH between May 2019 and March 2021.

### Study procedure, data collection and management

Demographic and clinical characteristics of patients are routinely collected by nurses and clinicians onto standardized admission report forms and then electronically entered on to an Excel database by trained staff. As per routine practice, patients with suspected sepsis were identified based on clinician suspicion, and appropriate samples including, urine, blood, pus, CSF, sputum and stool collected for culture by trained laboratory technicians and clinicians. After collection, samples were labelled with a unique bar code and stored in conditions appropriate for the specimen type before being sent to an external microbiology laboratory (Dr Lal PathLabs Limited) at the end of the day. For the duration of the study, the Dr Lal PathLabs laboratory was accredited by the National Accreditation Board for Testing and Calibration Laboratories (NABL), an autonomous body under the Government of India. The clinical samples were processed at the laboratory following standardized procedures adopted from the CLSI guidelines. In the event of a positive culture, AST was performed using the Kirby Bauer disk diffusion technique where standard discs with specified concentrations are used to detect the resistance patterns of each isolate. The plates were incubated overnight, after which the zone inhibition diameter was measured in millimetres (mm). The zones were interpreted as susceptible, intermediate, or resistant according to CLSI. In cases where the same isolates were identified in patients from different samples, we only considered the first isolates for the antibiotic susceptibility pattern analysis.

### Data collection and analysis

The culture and AST data for 22 months (May 2019 to March 2021) from the electronic laboratory system generated reports on all isolated organisms at the Dr Lal PathLabs microbiology laboratory were anonymized and entered into a Microsoft Excel 2010 spreadsheet. The culture and AST database were merged with a pseudo-anonymized inpatient line list to add information on the demographic and clinical characteristics of the patients before being exported to the Statistical Package for Social Science (SPSS) version 23 for analysis. Electronic data were regularly monitored and cross-checked with the source documents.

All data were described using descriptive statistics. Categorical variables including the baseline patient characteristics were described using numbers and percentages. Bacterial species antibiotic susceptibility patterns were presented as percentage of susceptibility (S) of total tested isolates. Susceptibility testing data were not reported for the specific antibiotic when an organism has intrinsic resistance to that antibiotic.

### Ethics

This was a retrospective study that used routinely collected programme data. No personal identifiers were used. This research was reviewed by and fulfilled the exemption criteria set by the Médecins Sans Frontières Ethics Review Board for *a posteriori* analysis of routinely collected clinical data and thus did not require MSF ERB approval (MSF ERB decision 2252). It was conducted with permission from the Medical Director Operational Center Barcelona Médecins Sans Frontières (MSF OCBA).

## Results

During the period of the study, a total of 1586 clinical specimens were sent for culture and AST from 577 AHD patients with suspected infections, with a median (IQR) of 2 (1–4) specimens per patient. The median (IQR) time between sample collection and results reporting was 4 (3–6) days, reflecting the transit time for samples to reach Delhi where they were processed.

The vast majority (*n* = 433; 75%) of AHD patients were male and the median (IQR) age was 38 (31–45) years. Even though most (*n* = 471; 81%) patients were on ART at the time of admission, 538 (94%) were admitted with WHO stage III or IV disease. More than half (*n* = 310; 54%) of patients had a CD4 count of less than 100 cells/mm^3^, and 133 (23%) patients had a CD4 count between 100 and 200 cells/mm^3^. The majority of patients had one or more comorbidity (Table 1). The most common comorbidities were TB (67%), *Pneumocystis* pneumonia (PCP) (14%), bacterial pneumonia (13%), *Cryptococcus* meningitis (12%) and visceral leishmaniasis (6%).

**Table 1.** Demographic and clinical characteristics of patients admitted with AHD

Characteristics	*n*	%
Age group (years)		
<15	17	2.9
15 to <30	87	15.1
30 to <45	306	53.0
45 to <60	150	26.0
≥60	16	2.8
Missing data	1	0.2
Sex		
Female	144	25.0
Male	433	75.0
ART status		
ART naive	105	18.2
ART experienced	471	81.6
Missing data	1	0.2
WHO stage		
I	3	0.5
II	14	2.4
III	108	18.7
IV	430	74.5
Missing data	22	3.8
CD4 group (cells/mm^3^)		
<100	310	53.7
100 to <200	133	23.1
≥200	87	15.1
Missing data	47	8.1
TB		
No	190	32.9
Yes	387	67.1
CrAg positive/cryptococcal meningitis		
No	508	88.0
Yes	69	12.0
Visceral leishmaniasis		
No	541	93.8
Yes	36	6.2
*Pneumocystis* pneumonia		
No	496	86.0
Yes	81	14.0
Bacterial pneumonia		
No	501	86.8
Yes	76	13.2

**Figure 1.**
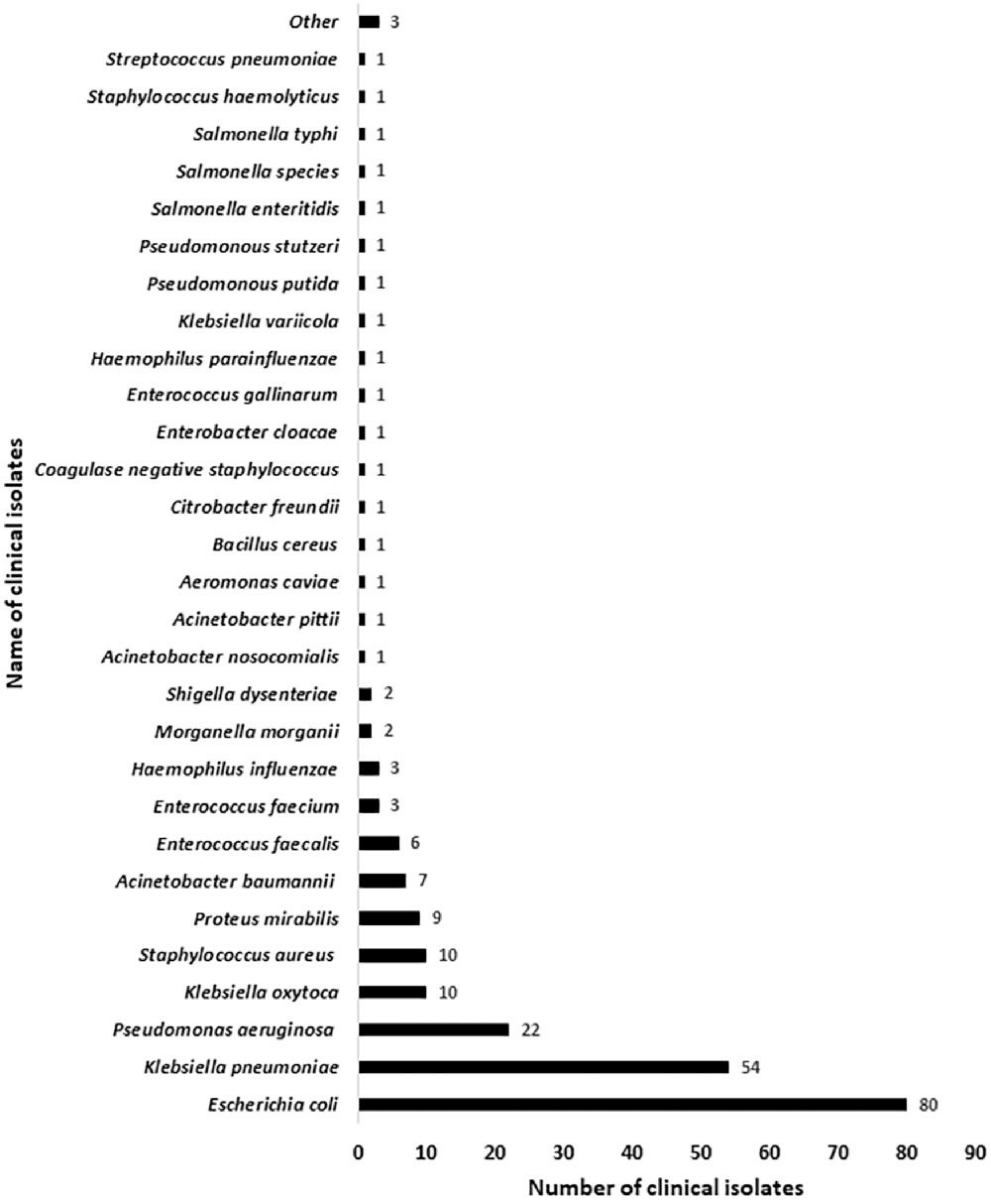
Prevalence of clinical isolates among collected clinical samples.

Table S1 (available as Supplementary data at *JAC-AMR* Online) shows the bacterial growth by the type of samples. In comparison with blood (3.3%; 20/600), largest yields were observed from urine (22.2%; 59/266), sputum (56.2%; 100/178) and pus (77.3%; 34/44). Pathogens isolated from stool and CSF samples showed lower yields of 2.9% (7/240) and 2.1% (3/144), respectively. Figure 1 shows the prevalence of Gram-negative bacteria, with the most common being *Escherichia coli* (13.9%; 80/577), followed by *Klebsiella pneumoniae* (9.4%; 54/577), *Klebsiella oxytoca* (1.7%; 10/577), *Pseudomonas aeruginosa* (3.8%; 22/577), *Proteus mirabilis* (1.6%; 9/577) and *Acinetobacter baumannii* (1.2%; 7/577). The overall susceptibility pattern of Gram-negative bacteria to the AST antibiotics is presented in Table 2. Tables S2 and S3 show the susceptibility patterns of Gram-negative bacteria to the AST antibiotics in sputum and urine samples, respectively.

**Table 2.** Antibiotic susceptibility patterns of Gram-negative bacteria from collected clinical samples

			Antibiotic																											
Bacterial Isolates	Total pathogens isolated		Gentamicin	Amikacin	Tobramycin	Netilmicin	Meropenem	Imipenem	Ertapenem	Ceftriaxone	Cefepime	Cefuroxime	Cefixime	Cefoperazone/sulbactam	Cefotaxime	Ampicillin/sulbactam	Ceftazidime	Amoxicillin/clavulanic acid	Piperacillin/tazobactam	Ampicillin	Ciprofloxacin	Norfloxacin	Levofloxacin	Ofloxacin	Co-trimoxazole	Colistin	Aztreonam	Nitrofurantoin	Chloramphenicol	Tigecycline
*E. coli*	80	S	60	82.5	68	93.8	62.5	60	64.4	11.3	20	8.9	9.7	35.5	13.6	11.5	0	28.8	43.8	6.8	6.3	2.9	6.9	4.8	10.4	28.8	13.8	71.4	75	100
		*N*	80	80	25	16	80	80	59	80	80	79	31	31	44	26	1	80	80	59	80	34	29	21	77	59	29	21	4	4
*K. pneumoniae*	54	S	51.9	63	36.8	44.4	50.9	53.7	56.4	5.7	27.8	3.8	5.3	21.1	6.5	5		20.4	40.7	R	14.8	11.8	5.3	6.7	18.8	20.5	15.8	20	50	33.3
		*N*	54	54	19	9	53	54	39	53	54	53	19	19	31	20		54	54		54	17	19	15	48	39	19	15	2	3
*K. oxytoca*	10	S	50	90	40	85.7	40	20		0	0	0	0	22.2	0	0		10	10	R	0	0	0	0	14.3		0	50		
		*N*	10	10	10	7	10	10		10	10	10	10	9	1	10		10	10		10	10	10	10	7		10	10		
*P. aeruginosa*	22	S	86.4	81.8	66.7		77.3	77.3	R	R	77.3			100	R		77.3	R	68.2	R	54.5		54.5		R	36.4	66.7	R	R	R
		*N*	22	22	3		22	22			22			1			22		22		22		22			22	9			
*P. mirabilis*	9	S	44.4	77.8	75	75	100	44.4	100	22.2	44.4	33.3	0	80	25	50		66.7	88.9	0	33.3	20	50	25	22.2	0	75	25		R
		*N*	9	9	4	4	9	9	5	9	9	9	5	5	4	4		9	9	5	9	5	4	4	9	3	4	4		
*A. baumannii*	7	S	14.3	42.9			0	0	R		16.7			0			0	R	0	R	14.3		14.3		14.3	42.9	R		R	100
		*N*	7	7			7	7			6			2			7		7		7		7		7	7				4

S, susceptibility percentage; *N*, number of isolates tested; R, intrinsically resistant. Empty cells reflect where an isolate was not tested against the associated antibiotic.

*E. coli* isolates showed very low susceptibility to commonly used antibiotics like cefotaxime (13%), ceftriaxone (11%), ciprofloxacin (6%), levofloxacin (7%) and amoxicillin/clavulanic acid (29%), whereas comparatively better susceptibility was observed to meropenem (62%), gentamicin (60%) and piperacillin/tazobactam (44%). Susceptibility remained high with amikacin (82%), netilmicin (94%) and nitrofurantoin (71%). Similarly, *K. pneumoniae* showed low susceptibility to frequently prescribed antibiotics such as colistin (20%), tigecycline (33%), nitrofurantoin (20%) and ceftriaxone (6%), in contrast to moderate susceptibility to piperacillin/tazobactam (41%), gentamicin (52%) and amikacin (63%). A similar pattern was observed in isolates of *K. oxytoca*, *P. aeruginosa*, *P. mirabilis* and *A. baumannii.* Co-trimoxazole, which is the most common prophylactic antibiotic to reduce the chances of PCP among HIV patients was also associated with very low (22%) susceptibility.

## Discussion

To the best of our knowledge, this is the first study from India that describes the AST pattern in AHD patients. One of the major causes of mortality in AHD patients is severe bacterial infections, which include major organ system infections such as bacterial pneumonia, isolated bacteraemia and severe diarrhoea.^12^ A study showed that among adult PLHIV, AIDS-related illnesses (46%) and bacterial infections (31%) were the leading causes of hospital admission after TB.^13^ Our study found similar patient characteristics in this cohort of patients, with concomitant TB and other AIDS-defining illnesses the most frequent reasons for admission. A similar picture has been described in studies addressing AHD and mortality in the global HIV programme.^12^

In our study, 77% of patients had CD4 counts less than 100 cells/mm^3^, and the majority of patients were coinfected with bacterial and opportunistic infections. Similar to the experiences in other AHD specialist wards, the majority (82%) in this study were already established on ART at the time of admission.^4^ For example, the MSF-supported AHD programme in Kinshasa, DRC showed that 71% of hospitalized AHD patients were established on ART prior to admission. The reasons for progression to AHD are multifactorial, reflecting health system factors (stockouts of ART, lack of prophylactic drugs, delayed detection of treatment failure) and individual factors (lack of means of transport, self-stigmatization, lack of knowledge about treatment).^4,14^

Gram-negative bacteria predominated in this study, with *E.coli* being the most common, followed by *K. pneumoniae*, *P. aeruginosa*, *K. oxytoca*, *P. mirabilis* and *A. baumannii*. A similar prevalence was found in two studies of PLHIV from south India and Bihar.^15,16^ The highest bacterial growth rates were found from sputum, followed by urine, pus and rectal swabs. The lowest (3.3%) bacterial growth was found from the blood samples.

ESBL susceptibility rates between 10% and 60% have recently been reported in India from diverse centres.^8^ When treated with ceftriaxone, ceftazidime or cefixime, patients with ESBL-producing *E. coli*, *Klebsiella* spp. and *P. mirabilis* showed poor treatment outcomes. The emergence of these infections poses a further emerging challenge and has become a major cause of morbidity and mortality in immune-compromised patients.^17^ In contrast to Iweriebor *et al.*’s^18^ finding of high susceptibility to the second-generation cephalosporins, our study showed a high level of resistance to co-trimoxazole, different generations of cephalosporins, and fluoroquinolones such as ciprofloxacin and levofloxacin. The extensive resistance to cephalosporins, particularly for *E. coli* isolates, is comparable to a recent study on Global Antimicrobial Resistance Surveillance System (GLASS) priority pathogens from India.^8^

*K. pneumoniae* also exhibited high levels of resistance against commonly prescribed antibiotics such as ceftriaxone, piperacillin/tazobactam, colistin, aztreonam and nitrofurantoin. Antibiotics such as amikacin, netilmicin, gentamicin and nitrofurantoin were associated with comparatively better susceptibility. Similarly, *K. oxytoca* showed high susceptibility to the antibiotics amikacin, netilmicin, gentamicin and nitrofurantoin, whereas 40% susceptibility to meropenem and only 10% susceptibility to piperacillin/tazobactam. In general, A*. baumannii* has an aminoglycoside, colistin and carbapenem resistance gene.^8^ Concerningly, co-trimoxazole was associated with susceptibility below 25% in all the Gram-negative bacteria. Overall, our study showed a similar susceptibility pattern in GLASS priority pathogens as reported in the literature.^8,16,19^ However, we were expecting substantially more resistant pathogens in our cohort of patients with AHD. This underestimation may be related to the small sample size, reflecting that it was based on a single ward.

Our research has several limitations. One major limitation of the study is lack of a control group of bacterial isolates from patients without HIV. Another limitation is that patients in our cohort frequently used antibiotics, including anti-TB medications, before presenting at the MSF ward and, on some occasions, culture samples were taken after starting antibiotics, which could affect the culture growth.

This study provided evidence that common Gram-negative bacteria are highly resistant to commonly used antibiotics in AHD patients, making treatment of these infections challenging and increasing both morbidity and cost of care. Timely identification of these resistant isolates and choosing the right antibiotics for empirical therapy with the use of regularly updated antibiograms may aid in clinical decision-making and considerably reduce mortality and prolonged hospital stays. The study findings are perhaps a first step in further research into improving care for this vulnerable population from an AMR perspective, which will assist in developing antibiotic use recommendations for patients with AHD. To catalyse this process, improved infection prevention and control practices, access to diagnostic tests and, critically, availability of quality-assured laboratories able to give quick and reliable results need to be prioritized in settings where AHD patients are treated. Within the inpatient setting, antibiotic stewardship practices need to be established with enhance surveillance and improved management for these patients.

## Supplementary Material

dlad151_Supplementary_Data

## Supplementary data

Tables S1 to S3 are available as Supplementary data at *JAC-AMR* Online.
