# Supplementary material for: Antibiotic susceptibility patterns of pathogens isolated from hospitalized patients with advanced HIV disease (AHD) in Bihar, India
Source: JAC Antimicrob Resist. 2024 Jan 2;6(1):dlad151. doi: 10.1093/jacamr/dlad151 (PMC10759003; doi:10.1093/jacamr/dlad151)
Supplement: dlad151_Supplementary_Data [file dlad151_supplementary_data.docx]

Supplementary Table 1: Prevalence of clinical isolates among collected clinical samples.

|  |  | Positive growth | |
| --- | --- | --- | --- |
| Type of samples | **Total samples** | **N** | **%** |
| Blood | 600 | 20 | 3.3% |
| Urine | 266 | 59 | 22.2% |
| Stool | 240 | 7 | 2.9% |
| Sputum | 178 | 100 | 56.2% |
| Cerebrospinal fluid | 144 | 3 | 2.1% |
| Pus | 44 | 34 | 77.3% |
| Rectal swab | 39 | 37 | 94.9% |
| Skin lesion | 33 | 3 | 9.1% |
| Miscellaneous | 18 | 5 | 27.8% |
| Pleural fluid | 10 | 1 | 10.0% |
| Ear Swab | 6 | 2 | 33.3% |
| Vaginal swab | 2 | 2 | 100.0% |
| Throat swab | 1 | 0 | 0.0% |
| Other | 5 | 2 | 40.0% |

Supplementary table 2: Antibiotics susceptibility patterns of gram-negative bacteria in Sputum samples.^1^

| **Bacterial Isolates** | **Total pathogens isolated** |  | **Gentamicin** | **Amikacin** | **Tobramycin** | **Meropenem** | **Imipenem** | **Ertapenem** | **Ceftriaxone** | **Cefepime** | **Cefuroxime** | **Cefixime** | **Cefoperazone/Sulbactam** | **Cefotaxime** | **Ampicillin Sulbactam** | **Ceftazidime** | **Amoxicillin clavulanic acid** | **Piperacillin tazobactam** | **Ciprofloxacin** | **Levofloxacin** | **co-trimoxazole** | **Colistin** | **Aztreonam** |
| --- | --- | --- | --- | --- | --- | --- | --- | --- | --- | --- | --- | --- | --- | --- | --- | --- | --- | --- | --- | --- | --- | --- | --- |
| ***Escherichia coli*** | **17** | **S** | **47.1** | **76.5** | **100** | **58.8** | **58.8** | **58.8** | **5.88** | **11.8** | **5.9** | **0** | **100** | **6.67** | **0** |  | **35.3** | **35.3** | **0** | **0** | **0** | **5.88** | **0** |
|  |  | **N** | 17 | 17 | 1 | 17 | 17 | 17 | 17 | 17 | 17 | 1 | 1 | 15 | 1 |  | 17 | 17 | 17 | 1 | 17 | 17 | 1 |
| ***Klebsiella pneumoniae*** | **27** | **S** | **51.9** | **66.7** | **50** | **51.9** | **51.9** | **51.9** | **0** | **40.7** | **0** | **33.3** | **66.7** | **0** | **0** |  | **29.6** | **44.4** | **14.8** | **0** | **11.1** | **25.9** | **25** |
|  |  | **N** | 27 | 27 | 4 | 27 | 27 | 27 | 26 | 27 | 27 | 3 | 3 | 20 | 4 |  | 27 | 27 | 27 | 4 | 27 | 27 | 4 |
| ***Pseudomonas aeruginosa*** | **20** | **S** | **85** | **85** | **100** | **80** | **80** | **R** | **R** | **80** |  |  | **100** | **R** |  | **85** | **R** | **75** | **60** | **60** | **0** | **30** | **71.4** |
|  |  | **N** | 20 | 20 | 2 | 20 | 20 |  |  | 20 |  |  | 1 |  |  | 20 |  | 20 | 20 | 20 | 0 | 20 | 7 |
| ***Acinetobacter baumannii*** | **3** | **S** | **0** | **33.3** |  | **0** | **0** | **R** |  | **0** |  |  | **0** |  |  | **0** | **R** | **0** | **0** | **0** | **0** | **33.3** | **0** |
|  |  | **N** | 3 | 3 |  | 3 | 3 |  |  | 3 |  |  | 0 |  |  | 3 |  | 3 | 3 | 3 | 3 | 3 | 0 |

S: Susceptibility percentage; N: Number of Isolates; R: intrinsically resistant

^1^Empty cells reflect where an isolate was not tested against the associated antibiotic.

Supplementary table 3: Antibiotics susceptibility patterns of gram-negative bacteria in Urine samples.^1^

| **Bacterial Isolates** | **Total pathogens isolated** |  | **Gentamicin** | **Amikacin** | **Tobramycin** | **Netilmicin** | **Meropenem** | **Imipenem** | **Ceftriaxone** | **Cefepime** | **Cefuroxime** | **Cefixime** | **Cefoperazone/Sulbactam** | **Ampicillin Sulbactam** | **Amoxicillin clavulanic acid** | **Piperacillin tazobactam** | **Ciprofloxacin** | **Norfloxacin** | **Levofloxacin** | **Ofloxacin** | **co-trimoxazole** | **Aztreonam** | **Nitrofurantoin** |
| --- | --- | --- | --- | --- | --- | --- | --- | --- | --- | --- | --- | --- | --- | --- | --- | --- | --- | --- | --- | --- | --- | --- | --- |
| ***Escherichia coli*** | **18** | **S** | **55.6** | **88.9** | **61.1** | **92.3** | **50** | **44.4** | **0** | **0** | **0** | **0** | **16.7** | **11.1** | **27.8** | **22.2** | **0** | **0** | **0** | **0** | **6.67** | **5.56** | **66.7** |
|  |  | **N** | 18 | 18 | 18 | 13 | 18 | 18 | 18 | 18 | 17 | 18 | 18 | 18 | 18 | 18 | 18 | 18 | 18 | 18 | 15 | 18 | 18 |
| ***Klebsiella pneumoniae*** | **15** | **S** | **40** | **40** | **33.3** | **44.4** | **35.7** | **40** | **6.67** | **0** | **0** | **0** | **13.3** | **6.67** | **6.67** | **20** | **6.67** | **6.67** | **6.67** | **6.67** | **22.2** | **13.3** | **20** |
|  |  | **N** | 15 | 15 | 15 | 9 | 14 | 15 | 15 | 15 | 14 | 15 | 15 | 15 | 15 | 15 | 15 | 15 | 15 | 15 | 9 | 15 | 15 |
| ***Klebsiella oxytoca*** | **10** | **S** | **50** | **90** | **40** | **85.7** | **40** | **20** | **0** | **0** | **0** | **0** | **22.2** | **0** | **10** | **10** | **0** | **0** | **0** | **0** | **14.3** | **0** | **50** |
|  |  | **N** | 10 | 10 | 10 | 7 | 10 | 10 | 10 | 10 | 10 | 10 | 9 | 10 | 10 | 10 | 10 | 10 | 10 | 10 | 7 | 10 | 10 |
| ***Proteus mirabilis*** | **4** | **S** | **75** | **75** | **75** | **75** | **100** | **100** | **25** | **50** | **50** | **0** | **100** | **50** | **50** | **100** | **50** | **25** | **50** | **25** | **50** | **75** | **25** |
|  |  | **N** | 4 | 4 | 4 | 4 | 4 | 4 | 4 | 4 | 4 | 4 | 4 | 4 | 4 | 4 | 4 | 4 | 4 | 4 | 4 | 4 | 4 |

S: Susceptibility percentage; N: Number of Isolates; R: intrinsically resistant

^1^Empty cells reflect where an isolate was not tested against the associated antibiotic.
